# Effects of brain microRNAs in cognitive trajectory and Alzheimer’s disease

**DOI:** 10.1002/alz.087046

**Published:** 2025-01-03

**Authors:** Selina M. Vattathil, David A. Bennett, Julie A. Schneider, Aliza P. Wingo, Thomas S. Wingo

**Affiliations:** ^1^ Emory University School of Medicine, Atlanta, GA USA; ^2^ Rush University Medical Center, Chicago, IL USA; ^3^ Atlanta VA Medical Center, Decatur, GA USA; ^4^ Emory University, Atlanta, GA USA

## Abstract

**Background:**

microRNAs (miRNAs) are small RNAs involved in regulating gene expression by repressing target protein‐coding genes. Hundreds of miRNAs are expressed in human brain, but our understanding of their role in Alzheimer’s disease (AD) and cognitive decline is limited.

**Method:**

We performed miRNA differential expression analysis using small RNA sequencing data generated from dorsolateral prefrontal cortex samples from 641 participants of the Religious Orders Study (ROS) and Memory and Aging Project (MAP). After quality control, 528 miRNAs were available to test for differential expression in cognitive trajectory (for five cognitive subdomains and global cognition), amyloid, tangles, and AD clinical diagnosis. Initial analyses were performed using association models that included adjustment for technical and demographic covariates. To identify miRNAs associated with AD‐related outcomes independently of co‐occurring age‐related cerebral pathologies, secondary analyses were conducted using models augmented to also adjust for the pathologies. Sex interaction analyses were performed to identify sex‐biased miRNAs, and Mendelian randomization was conducted to assess the evidence that genetically‐regulated miRNA abundance mediates the observed trait associations.

**Result:**

We identified 311 differentially expressed miRNAs for AD, its hallmark pathologies, or cognitive trajectory. Our secondary analysis identified 137 miRNAs that were associated with AD‐related outcomes independently of up to ten age‐related cerebral pathologies. The protein‐coding genes predicted to be repressed by the differentially expressed miRNAs were enriched in lipoproteins, transcription, postsynaptic signaling, and cellular senescence. Sex interaction analysis identified 5 miRNAs with different observed associations in males and females. Finally, Mendelian randomization identified 15 miRNAs with evidence that their expression roles play a causal role in the associated trait.

**Conclusion:**

We find evidence for some miRNAs being associated with AD‐related traits and cognitive trajectory through age‐related cerebral pathologies while other miRNAs are independently associated with AD‐related outcomes. The miRNAs consistent with a causal role are promising candidates for future studies in model systems to understand how miRNAs contribute to AD and cognitive decline.

